# Port-Based Anonymous Communication Network: An Efficient and Secure Anonymous Communication Network

**DOI:** 10.3390/s23218810

**Published:** 2023-10-29

**Authors:** Xiance Meng, Mangui Liang

**Affiliations:** 1 Institute of Information Science, Beijing Jiaotong University, Beijing 100044, China; mengxiance@bjtu.edu.cn; 2 Beijing Key Laboratory of Advanced Information Science and Network Technology, Beijing Jiaotong University, Beijing 100044, China

**Keywords:** anonymous communication networks, anonymity, routing, Tor

## Abstract

With the rise of the internet, there has been an increasing focus on user anonymity. Anonymous communication networks (ACNs) aim to protect the identity privacy of users in the network. As a typical ACN, Tor achieves user anonymity by relaying user data through a series of relay nodes. However, this results in higher latency due to the transmission of network traffic between multiple nodes. This paper proposes a port-based anonymous communication network (PBACN) to address this issue. First, we propose a path construction algorithm. This algorithm describes constructing paths by partitioning the communication path information, which can reduce the probability of being discovered by adversaries. Secondly, we design a port-based source routing addressing method. During data transmission from the source to the destination, each node can directly forward the data by resolving the address into the port of each node. This method eliminates the need for table lookups, reducing the complexity of routing. Lastly, we propose an entropy-based metric to measure the anonymity of different ACNs. In terms of experimental evaluation, we quantitatively analyze the anonymity and end-to-end delay of various ACNs. The experimental results show that our proposed method reduces end-to-end delay by approximately 25% compared to Tor. When the adversary fraction is 20%, PBACN can improve the anonymity degree by approximately 4%.

## 1. Introduction

In the internet era, privacy protection and anonymity are becoming increasingly important [1]. Anonymous communication technology is essential for privacy protection, allowing users to communicate anonymously online and avoid surveillance and tracking. However, traditional anonymous communication schemes have high latency and poor anonymity. Therefore, designing an efficient and secure anonymous communication scheme is necessary.

DC-nets (Dining Cryptographers Networks) [2] allow users to exchange messages without revealing their identities. Each user encrypts the message using a secret key and sends it to all other users in the network [3]. Then, the members use a shared key that only they know to decrypt the message. Mix-net uses a series of nodes to confuse and forward messages, making tracing the message’s source difficult. Each node in the network only knows the previous and next nodes in the chain and cannot link the message to its sender or receiver. However, these methods are generally plagued by problems such as high latency and poor scalability.

The P2P network [4,5,6] can also improve anonymous communication, in which users communicate directly without a central server. It can improve scalability and resilience but may also introduce new security risks, such as the possibility of Sybil attacks [7].

Dovetail [8] is an anonymous communication network based on the next-generation internet routing protocol. It provides anonymity against active attackers but still struggles to cope with traffic analysis attacks [9,10,11].

We aim to construct an anonymous communication method to achieve low-latency transmission and ensure anonymity. A common practice is to use relay routers to encrypt the data passing through and hide the actual information of the packets to improve anonymity. In addition to the delays caused by encryption and decryption, each relay router’s transmission time will also increase relatively due to the longer transmission path and more nodes passing through. There are two main methods to reduce end-to-end transmission delay: one is to select nodes with high bandwidth or geographical distance preferentially  [12,13] during the relay selection phase to minimize delay in the transmission process; the other is to reduce the number of relay nodes to reduce the transmission path. However, both methods will result in loss of anonymity.

The routing type used in Tor is called hop-by-hop routing. In Tor, each relay node decrypts the message to obtain the IP address of the next hop, and the communication process involves multiple routing lookups. The advantages of the source routing [14] protocol are as follows: the network topology is simpler, and there is no need to maintain complex routing tables, which can avoid data packet loss caused by routing loops and can calculate the shortest path faster, improving routing efficiency. In the case of expanding network scale, source routing can also maintain good scalability. Based on this, we designed an anonymous communication network based on port forwarding. During the routing process, intermediate nodes can directly parse the address into port numbers and forward the data through the corresponding ports. This method eliminates the need for table lookups, thus reducing the complexity of the switch. The reduction in switch complexity is beneficial for energy conservation, which also extends the network lifespan [15].

We evaluated the performance of PBACN and compared it with traditional Tor routing strategies. Due to the mechanism of anonymous communication networks, high latency has always been a common issue. Lower latency in anonymous communication networks will attract more users to join. The experimental results show that PBACN can provide better performance than other routing strategies.

We also analyzed the anonymity of PBACN. Anonymity is the most essential characteristic of anonymous communication networks. Higher anonymity means a higher probability that users will go unnoticed by adversaries in the network. The experimental results show that PBACN can improve anonymity, reduce the success rate of attackers, and thus increase user privacy and security.

The organizational structure of this article is as follows. In Section 1, we introduce the research background of this article. In Section 2, we present the related work. Section 3 introduces the design ideas and technical implementation of PBACN. We describe the performance evaluation of PBACN and compare it with traditional Tor routing strategies in Section 4. Then, in Section 5, we analyze the anonymity of PBACN. Finally, we summarize this article’s work and propose future research directions.

## 2. Related Works

This section will introduce two research fields directly related to our problem: source-controlled routing protocols and network-layer anonymity protocols.

### 2.1. Source-Controlled Routing Protocols

Source-controlled routing protocols [16,17] are an essential topic for the next-generation internet routing scheme. The information carried in the packets by the initiator controls the routing information of data packets. This method of controlling routing information at the source has robustness and flexibility. It also has benefits because intermediate routers cannot obtain complete path information.

Our work is based on a new type of network addressing method called Vector Network (VN) [18,19]. VN is a source-controlled routing method in which each network node has a particular data-forwarding capability. When the source sends a data packet, the source node stores the sequence of the path in the packet header, and the length of each path segment is related to the number of ports on the node passed through. When the data pass through each node, it will extract the corresponding port number and forward data to that port. At the same time, the extracted port number will also be removed from the path sequence. Since the source node defines the forwarding path of the data, the intermediate nodes do not need to query the routing table again during the data transmission process, thereby enhancing the robustness and flexibility of the network.

### 2.2. Low-Latency Anonymous Communication Systems

Some existing research has proposed low-latency anonymous communication schemes based on different routing strategies to meet the needs of some interactive applications, such as web browsing and instant messaging.

Tor can effectively protect users’ identity and privacy, allowing users to be free from internet surveillance and tracking. When using Tor, users’ network traffic is encrypted and transmitted. Each relay node can only decrypt the information of its next node, and so on, until the final node sends the information to the destination server. In this process, each node only knows the information of the previous and next nodes and does not know the source or destination of the data. It protects the user’s IP address and location, thereby protecting their identity and privacy. Since Tor uses multi-layer encryption, it can protect user data from being stolen or tampered with. And because each node only knows the information of the previous and next nodes, even if a node is attacked or monitored, it cannot see the user’s real identity and location.

HORNET [20] is a low-latency onion routing system implemented based on the next-generation network architecture. In HORNET, intermediate nodes only need to perform symmetric encryption on the packets. The sender establishes keys with each node along the path during the establishment process. Then, the sender embeds these keys and routing information into the packet header for transmission, thus achieving high scalability. Due to the packet header reused in HORNET, it cannot prevent replay attacks. So attackers can modify packets at will, making it difficult for users to distinguish between modified packets and legitimate packets. Adversaries can insert identifiable fingerprints in the traffic, which helps to de-anonymize the sender. Lightweight anonymous communication systems like LAP [21] and Dovetail [8] defend against topology attacks by encrypting routing information in the packet header. However, in both schemes, the packets remain unchanged during the transmission between hops, allowing adversaries to de-anonymize communication links by analyzing the correlation between packages at different nodes. TARANET [22] adopts end-to-end traffic shaping and packet fragmentation techniques to achieve anonymity at the network layer. It can even defend against active attacks but incurs specific latency.

T-hybrid [23] is a hybrid routing scheme that uses source routing between groups and hop-by-hop routing within groups. It combines mix-nets [24] with TPKE (Threshold Public-Key Encryption) [25] for better key management. The source selects multiple groups to generate the onion and encrypts by TPKE. Each receiving node generates its decryption share in each group and attaches it to the ciphertext. After the share number exceeds the threshold, the last node combines all shares and processes the onion. At the same time, symmetric encryption is used for each hop within the group. T-hybrid effectively combines onion routing with hop-by-hop routing, improving its resilience and increasing its latency by about 20%–25% compared to Sphinx [26].

As shown in Table 1, based on the comparison of existing research work, it is found that balancing anonymity and latency in anonymous communication networks is a challenging task. Taking Hornet as an example, although it has low latency, it faces challenges such as replay attacks. Compared to these anonymous communication networks, our designed port-based source routing addressing method can reduce routing complexity without affecting routing performance, thus achieving lower latency and ensuring anonymity.

## 3. Design

PBACN is an anonymous communication network based on port forwarding, which is efficient and has high anonymity. This section outlines the network model and describes the path construction and data packet-forwarding processes.

### 3.1. Network Model

PBACN consists of various nodes, including user nodes, web nodes, group leader nodes, and directory servers. Users are ordinary users who need anonymous access to the internet, web nodes are the standard websites accessed, and IN nodes are entry nodes in the relay group, which resolve the address assigned by the group leader and perform data forwarding. OUT nodes are the exit nodes in a relay group. They forward data to the next relay group’s IN or web nodes. Group leaders are the leader nodes in a relay group, also known as relay group leaders. They are responsible for finding paths between group leaders and the path from the IN nodes to the OUT nodes. The directory server maintains the information of relays and group leaders.

In this network model, users first request to download relay group leader information from the directory server. The directory server randomly selects a part of the online node information from the maintained relay group leader list and sends it to the user. The directory server only knows the data of each group leader. Each group leader only knows the routing information between groups and within the group and does not know the routing information of other groups. If an attacker attempts to destroy the directory server, we only provide partial network information to users, thus protecting the network’s anonymity.

### 3.2. Path Construction

In PBACN, path construction is relatively complex and requires a series of steps. The user first requests the relay group leader information from the directory server and obtains a random selection of online node information from the maintained relay group leader list. Next, the user must request inter-group paths and IN node to OUT node paths for each relay group leader. After the user selects the relay group leader and IN and OUT nodes, each group will generate paths between each node, using a source-controlled routing algorithm and feedback the path information to the sender, who will negotiate the key with each relay group leader, encrypt the data, and then transmit the encrypted data to the next node. The path construction process is described in Algorithm 1. Among them, the number of groups is *g*, and the list of the relay group leaders obtained by the user from the directory server is gl_list. gl[i] is the relay group leader randomly selected by the user from the list. Once the relay group leader information is determined, the user will sequentially request the address information addr[i] from the sender to the relay group leader and the destination. Finally, we can obtain the source-to-destination address all_addr by merging all the addr[i]. Assuming three groups and that the address addr[i] for each segment from the source to the destination is {21,34,12,11}. The address all_addr from the sender to the destination is {21341211}, which indicates the path from the sender to the destination.
**Algorithm 1** Path construction algorithm**Require:** A list of group leaders gl_list fetched from Directory Server, group number *g*;
**Ensure:** The address from the user to the website all_addr
 1:**for** i←0 to g−1 **do** 2:    gl[i]←choose_gl(gl_list) 3:**end for** 4:addr[0]←get_addr(user,gl[0]) 5:**for** i←0 to g−2 **do** 6:    addr[i+1]←get_addr(gl[i],gl[i+1]) 7:**end for** 8:addr[g]←get_addr(gl[g−1],website) 9:**for** i←0 to *g* **do** 10:    all_addr←all_addr+addr[i] 11:**end for** 12:**return** all_addr

**Figure 1:**
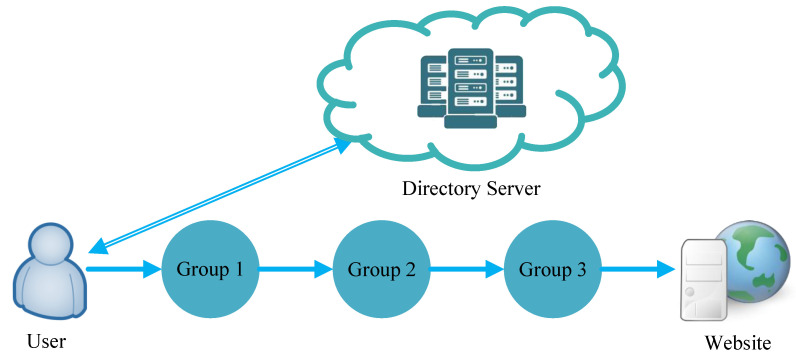
PBACN architecture with three groups.

Next, we will introduce the complete design of PBACN hierarchically. As shown in Figure 1, the user requests the relay group leader information from the directory server and accesses the website through data forwarding based on the obtained information.

**Figure 2:**
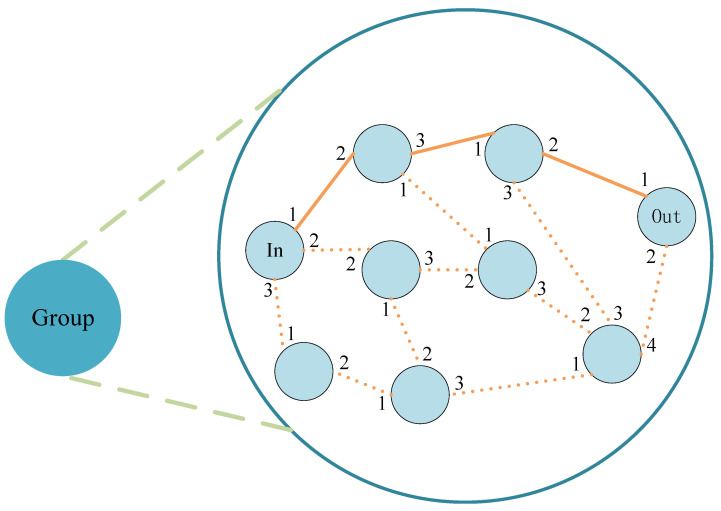
Network topology within the group.

In PBACN, the directory server can also register nodes and form groups. Any node with high bandwidth, online time, and routing capability can spontaneously register as a group leader. When the directory server receives an application, it will detect if there is a group leader online in the vicinity. The applying node will be registered as a group leader if there is none. Nodes near the group leader that are not part of any group will spontaneously query the directory server for nearby group leaders and join the group. Figure 2 shows the intragroup relationship diagram. After selecting IN and OUT nodes, IN nodes can access OUT nodes through the source routing method. As shown in the figure, after the message passes through the IN node, it can forward data through ports 1, 3, and 2 of each node because the source controls the path, so there is no need to use hop-by-hop routing, thereby saving routing time.

**Figure 3:**
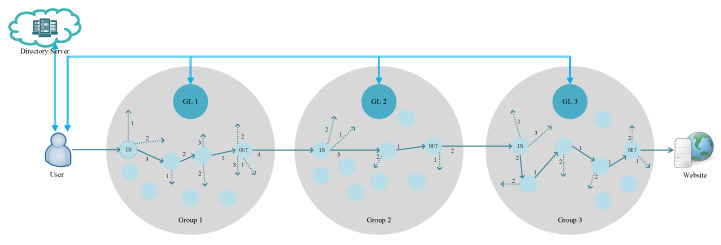
Overview of the PBACN.

Figure 3 shows the complete architecture. The sender requests relay group leader (GL) information from directory servers. Then, it requests path information between these relay group leaders. Taking GL 1 as an example, GL 1 will request path information from the IN node to the OUT node and OUT to subsequent IN nodes in GL 1 and reply to the sender. In addition, the sender will negotiate keys with GLs separately and encrypt the transmitted data to avoid eavesdropping by adversaries.

These technologies provide good path construction and message transmission guarantees, making PBACN an efficient, real-time, secure, and reliable anonymous communication network.

### 3.3. Data Forwarding

In PBACN, data forwarding is performed through relay nodes. When a user wants to send a message, the message is first encrypted and sent to the IN node of the first relay group. This node forwards the message in turn until the message reaches the OUT node of the last relay group. Each node decrypts and forwards the message to the next node, ensuring message security and privacy. At the same time, we use relay groups to segment the path information so that directory server nodes and relay group leaders cannot grasp the complete path information, thus protecting the sender’s privacy. In addition, source routing based on port forwarding is a very effective routing strategy in PBACN, which can significantly improve the performance of anonymous communication. The working principle of source routing is that when sending a message, the source node adds a set of routing information indicating how the message should reach the destination node. In the relay group, each node can directly parse the following hop address and forward the message to that address until the message reaches the target node location. Compared to the hop-by-hop routing method, this design reduces the table lookup time, as it does not require table lookups. In addition, this design separates the data plane from the control plane, with switches only responsible for data forwarding, and reducing the switches’ complexity.

## 4. Performance Evaluation

We designed simulation experiments to compare and evaluate our proposed method with some existing research work, proving that our method is superior in reducing end-to-end delay, especially in complex data processing and network congestion cases. In addition, we also conducted a detailed analysis of the comparative results, pointing out the critical impact of end-to-end delay on network performance and user experience. The simulation demonstrates that our research can make some improvements in enhancing network transmission efficiency.

### 4.1. Performance Metrics

End-to-end delay is the time required for a data packet to travel from the sender to the receiver. Evaluating end-to-end delay can help us understand the performance of different methods and improve network transmission efficiency and user experience. In this article, we evaluated the performance of our proposed method and existing research work. We proved that our method is superior in reducing end-to-end delay, especially in complex data processing and network congestion cases.

In Section 2.1, we mentioned the concept of VN. As a source-controlled routing method, nodes only need to parse the address sent by the previous node into a port number and forward it directly when forwarding data. The node does not need to perform table lookup routing, reducing the routing complexity. Compared with existing research, our method can more accurately determine the transmission path of data packets and decrease the delay cost of hop-by-hop routing.

### 4.2. Simulation Design

We used OMNeT++ [27] to evaluate the performance of different solutions through simulation. We compared three solutions: Tor, T-Hybrid, and the PBACN proposed in this article.

In this experiment, we followed the process below:

1. Construct a simulated real network: We extracted node information by processing the Consensus file, which contains information such as node bandwidth and online time. We set the link parameters between nodes to construct a simulated real network, and Different IDs identify different nodes. Because we want to compare different methods, we designed different node processing rules to correspond to different methods during simulation.

2. Communication: We randomly selected two nodes as the source and destination nodes. The source initiates a communication request to the directory, and the directory queries the address and sends it to the source end. The source end resolves the address and forwards it layer by layer. Compared with other methods, the port-forwarding-based anonymous communication network we constructed does not require hop-by-hop routing, saving the time consumption of hop-by-hop routing and avoiding information leakage during the routing process, which is undoubtedly essential for anonymous communication networks.

### 4.3. Results Analysis

Tor is the most popular and widely researched low-latency anonymous communication network, providing sender privacy for internet users. T-Hybrid is the latest anonymous communication network that combines onion mix-net with hop-by-hop routing, offering excellent resilience and anonymity. Therefore, we evaluate and compare with the end-to-end latency of Tor and T-Hybrid.

**Figure 4:**
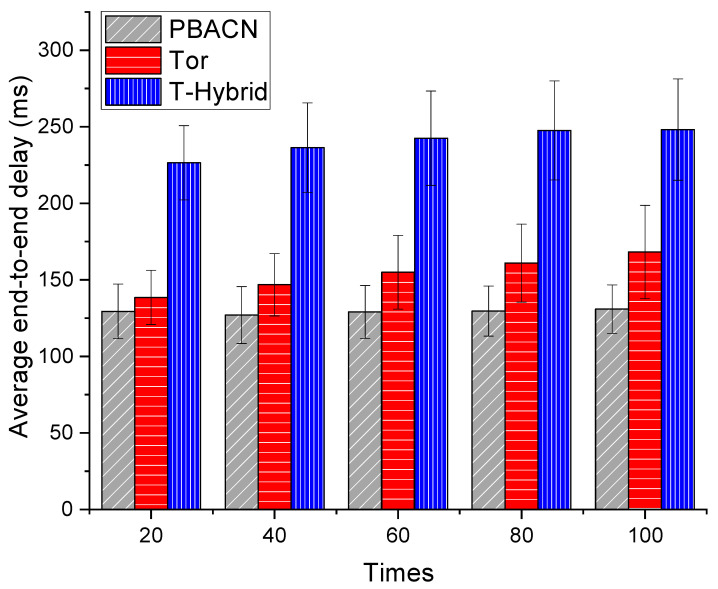
Comparison of average end-to-end delay results.

Figure 4 compares the average end-to-end delay results. We simulated Tor, T-Hybrid, and PBACN in OMNeT++ and deployed 100 nodes. The nodes have the same bandwidth, and the links have the same latency. In each experiment, the sender and receiver are randomly selected. We conducted 100 experiments and recorded the end-to-end latency of each method in each experiment. The end-to-end delay is the difference between the receiving and sending times. In every 20 experiments, we calculated the average end-to-end delay from the beginning to that moment. We used a histogram to present the experimental results, with the abscissa representing the number of experiments and the ordinate representing the average end-to-end delay for each corresponding experiment. We also added error bars to the graph to show that the data obtained are reliable because they exhibit minimal fluctuations. As shown in the diagram, we can observe that the end-to-end delay of PBACN is generally lower than other methods. Compared to Tor, our proposed method reduces the end-to-end delay by approximately 25%.

**Figure 5:**
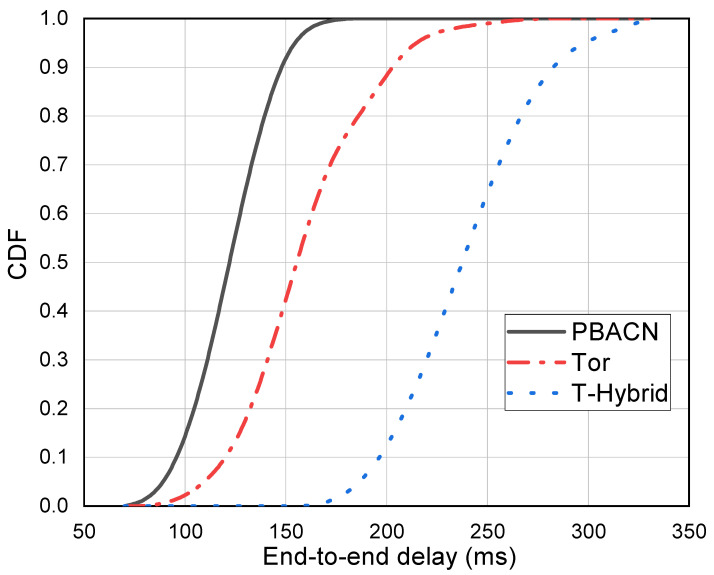
CDF under various end-to-end delays.

Figure 5 depicts different methods’ CDF (cumulative distribution function) under various end-to-end delays. The *x*-axis represents the end-to-end delay, and the *y*-axis represents the CDF. The curves in the graph indicate the proportion of end-to-end delay for different methods in different intervals. We can see that the CDF of our proposed PBACN can reach one faster, indicating that the end-to-end delay of PBACN is much lower than 180 ms, while the maximum end-to-end delay of other methods is higher than that of PBACN.

In summary, onion-based ACNs such as Tor have multiple relays that introduce additional latency in both the encryption and decryption processes and the hop-by-hop routing process. On the other hand, mix-based ACNs such as T-Hybrid combine hybrid routing with TPKE for improved key management. However, each group receiving the mix must collaborate with the sender for cooperative encryption, resulting in additional latency costs.

In contrast, our proposed PBACN first utilizes source-controlled routing, reducing routing time. Additionally, only the group leader must negotiate encryption with the sender, resulting in more saved encryption time than T-Hybrid. Therefore, PBACN has a lower end-to-end delay than other methods, which can provide users with a better experience and improve network performance and efficiency.

## 5. Anonymity Analysis

In this section, we introduce threat models and compare the anonymity of different anonymous communication networks.

### 5.1. Threat Model

As an anonymous communication network, while it provides anonymity to users, some malicious adversaries will inevitably come to disrupt its anonymity. To better deal with these vicious attacks, we need to define the adversary’s capabilities to analyze their threat better.

We use the threat model proposed by Syverson et al. [28] as the basis for the adversary. Taking Tor as an example, Tor’s entry node knows the client’s IP address in the anonymous communication network, while the exit node knows the server’s IP address. When an adversary controls these two nodes [29,30], they can use traffic analysis to confirm the communication relationship, thereby breaking the anonymity of the link.

We assume that the adversary can control a portion of the relay nodes. Secondly, since active adversaries are more likely to be discovered by users, the adversary cannot modify, delete, or delay traffic. The adversary can use the controlled nodes to monitor and analyze network traffic and the traffic of user requests and responses, thereby inferring the sender and receiver of the message and breaking anonymity. The model is also the most prevalent threat model faced by anonymous networks.

### 5.2. Anonymity Degree

A system can achieve maximum anonymity when an attacker assumes that all nodes in the anonymity set have an equal probability of being the sender of the message [31]. Thus, the probability distribution determines the anonymity degree. For a given probability distribution, the concept of entropy [32] in information theory provides a measure of information. Therefore, we can use entropy to calculate the anonymity of the system [33].

Let *N* be the number of nodes in the system and $p_{i}$ be the probability that each node is inferred as the sender by the adversary. We define H(N) as the maximum entropy of the system, which is:

(1) $$H\left(N\right)=-\sum_{i=1}^{N}p_{i}\log_{2}\left(p_{i}\right).$$

When the adversary reduces the anonymity set to *S* through an attack, the new entropy is H(S), and the information obtained by the adversary is H(N)−H(S). We use the maximum entropy H(N) to normalize this value, and therefore the anonymity degree is:

(2) $$d=1-\frac{H(N)-H(S)}{H(N)}=\frac{H(S)}{H(N)}.$$

If S=N, the adversary fails to reduce the anonymity set and d=1. The system has the maximum anonymity degree. When the adversary receives the sender’s identification, the system entropy is 0, and d=0. The system has the minimum anonymity degree. We can compare the anonymity degrees in different anonymity systems based on the above definition.

**In Tor:** According to our threat model, assuming the proportion of nodes controlled by the adversary is *f*, we analyze the system’s anonymity under different situations, where the probability of each scenario occurring is $q_{i}$ and the corresponding anonymity degree is $d_{i}$. The anonymity degree of the system is:

(3) $$D=\sum_{i=1}^{3}q_{i}*d_{i}.$$

(1) When the adversary controls the sender:

(4) $$q_{1}=f,$$

(5) $$d_{1}=0.$$

(2) When the adversary does not control the sender but controls both the entry and exit nodes of Tor:

(6) $$q_{2}=\left(1-f\right)*f^{2},$$

(7) $$d_{2}=0.$$

(3) When the adversary does not control the sender and neither the entry nor the exit nodes of Tor:

(8) $$q_{3}=\left(1-f\right)\left(1-f^{2}\right).$$

According to Equations (1) and (2), in this case:

(9) $$p_{i}=1/S=1/N\left(1-f\right),$$

(10) $$H\left(S\right)=\log_{2}\left(N\left(1-f\right)\right),$$

(11) $$d_{3}=\frac{\log_{2}\left(N\left(1-f\right)\right)}{\log_{2}\left(N\right)}.$$

Therefore, according to Equation (3), Tor’s anonymity degree is:

(12) $$D_{Tor}=\left(1-f\right)\left(1-f^{2}\right)\frac{\log_{2}\left(N\left(1-f\right)\right)}{\log_{2}\left(N\right)}.$$

**In T-Hybrid:** T-Hybrid consists of multiple groups, with an average group size of *g*. For one of these groups, the probability that at least one node is compromised is $1-{\left(1-f\right)}^{g}$.

We still consider three cases:

(1) When the adversary controls the sender:

(13) $$q_{1}=f,$$

(14) $$d_{1}=0.$$

(2) When the adversary does not control the sender, but at least one node in the first group and the last group of T-Hybrid are controlled by the adversary:

(15) $$q_{2}=\left(1-f\right)\left({\left(1-{\left(1-f\right)}^{g}\right)}^{2}\right),$$

(16) $$d_{2}=0.$$

(3) When the adversary does not control the sender, and at least one group is entirely uncontrolled by the adversary:

(17) $$q_{3}=\left(1-f\right)\left(2*{\left(1-f\right)}^{g}-{\left({\left(1-f\right)}^{2}\right)}^{g}\right),$$

(18) $$d_{3}=\frac{\log_{2}\left(N\left(1-f\right)\right)}{\log_{2}\left(N\right)}.$$

Therefore, the anonymity degree of T-hybrid is:

(19) $$D_{T\_hybrid}=\left(1-f\right)\left(2*{\left(1-f\right)}^{g}-{\left({\left(1-f\right)}^{2}\right)}^{g}\right)\frac{\log_{2}\left(N\left(1-f\right)\right)}{\log_{2}\left(N\right)}.$$

**In PBACN:** Our proposed method has multiple groups compared to Tor, and the first node of each group cannot directly obtain the address information of the previous node. Therefore, the adversary must control both the leader node and the group’s first node to compromise the anonymity. There are three cases:

(1) When the adversary controls the sender:

(20) $$q_{1}=f,$$

(21) $$d_{1}=0.$$

(2) When the adversary does not control the sender but controls the entry node of the first group and the exit node of the last group in PBACN, as well as the leaders of both groups:

(22) $$q_{2}=\left(1-f\right)f^{4},$$

(23) $$d_{2}=0.$$

(3) Other cases not mentioned above:

(24) $$q_{3}=\left(1-f\right)\left(1-f^{4}\right),$$

(25) $$d_{3}=\frac{\log_{2}\left(N\left(1-f\right)\right)}{\log_{2}\left(N\right)}.$$

Therefore, the anonymity degree of PBACN is:

(26) $$D_{PBACN}=\left(1-f\right)\left(1-f^{4}\right)\frac{\log_{2}\left(N\left(1-f\right)\right)}{\log_{2}\left(N\right)}.$$

In the PBACN we propose, multiple groups and leaders exist. Each group leader can only access a portion of the addresses from the source to the destination. Therefore, for an adversary to de-anonymize the sender’s identity, they must simultaneously control all group leaders and the first node of the first group. In contrast, if an adversary wants to de-anonymize the sender in Tor, they only need to control the entry and exit nodes simultaneously. Therefore, PBACN offers higher anonymity. The diagram can also demonstrate our conclusion.

**Figure 6:**
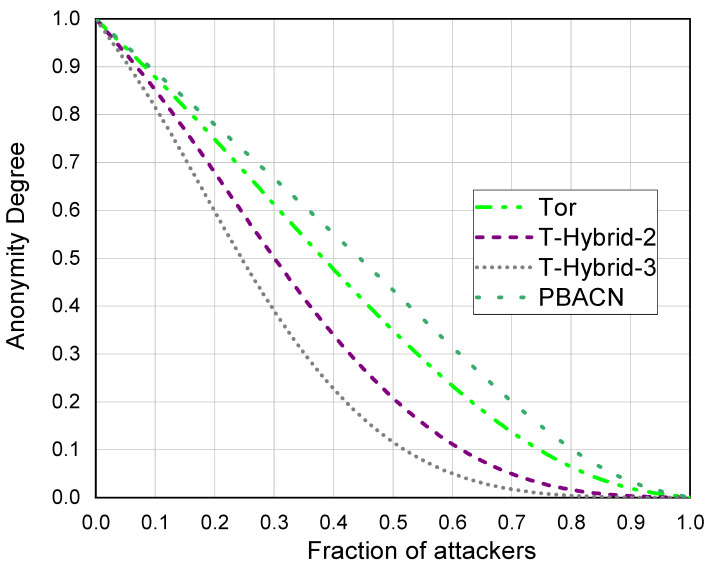
Anonymity degree.

As shown in Figure 6, according to Equations (12), (19), and (26), we compared the anonymity of different anonymous communication networks under varying fractions of attackers. For T-hybrid, we also compared the changes in group size. The diagram shows that when the fraction of attackers increases, the anonymity degree of the network decreases. When there are no attackers in the network, it has the highest anonymity degree. We found that, except when approaching the lowest and highest fraction of attackers, we can easily distinguish the anonymity degree of each curve. Therefore, our definition of anonymity degree effectively expresses the anonymity of different ACNs. We can see that PBACN has a higher anonymity degree than other methods. When the adversary fraction is 20%, PBACN can improve the anonymity degree by approximately 4%.

## 6. Conclusions

This paper proposes a port-based anonymous communication network called PBACN, which uses a source routing method based on port forwarding for rerouting. Compared with other anonymous communication networks, the PBACN can significantly reduce routing time while ensuring anonymity. The experimental results of this method show that it can dramatically improve the efficiency and anonymity of anonymous communication and is a feasible anonymous communication solution. In the implementation process, we improved the traditional routing strategy and proposed a new source routing method. The source routing method uses port forwarding to reroute messages, allowing messages for which the sender required hop-by-hop routing to reach the destination directly, thus reducing the routing time. The PBACN method can also ensure communication security and anonymity by improving the traditional routing strategy. In the experiment, we compared the PBACN method with the other anonymous communication networks, proving that it enhances communication efficiency while ensuring anonymity.

We will work on integrating the next-generation network with the existing network system in the future based on our current research. This work focuses on deploying our proposed methods in real networks and improving the transmission efficiency and anonymity of the network in practical applications. Additionally, ACN will encounter various attack methods and adversaries in practical applications. Therefore, we will analyze the characteristics of different adversary nodes and study a node selection strategy that can detect malicious nodes.

## Figures and Tables

**Table 1:** Comparison of anonymous communication systems.

ACNs	Routing Type	Latency	Challenge
Tor	hop by hop	middle	traffic analysis
LAP	source controlled	low	traffic analysis
Hornet	source controlled	low	replay attack
TARANET	source controlled	middle	increased latency
T-Hybrid	hybrid	middle	increased latency
Dovetail	source controlled	low	traffic analysis

## Data Availability

Not applicable.
